# Multi-Person Pose Estimation using an Orientation and Occlusion Aware Deep Learning Network

**DOI:** 10.3390/s20061593

**Published:** 2020-03-12

**Authors:** Yanlei Gu, Huiyang Zhang, Shunsuke Kamijo

**Affiliations:** 1 College of Information Science and Engineering, Ritsumeikan University, Kusatsu, Shiga 525-8577, Japan; 2 Institute of Industrial Science, The University of Tokyo, Tokyo 153-8505, Japan; zhanghuiyang@kmj.iis.u-tokyo.ac.jp (H.Z.); kamijo@iis.u-tokyo.ac.jp (S.K.)

**Keywords:** pose estimation, body orientation, multi-person, multi-task

## Abstract

Image based human behavior and activity understanding has been a hot topic in the field of computer vision and multimedia. As an important part, skeleton estimation, which is also called pose estimation, has attracted lots of interests. For pose estimation, most of the deep learning approaches mainly focus on the joint feature. However, the joint feature is not sufficient, especially when the image includes multi-person and the pose is occluded or not fully visible. This paper proposes a novel multi-task framework for the multi-person pose estimation. The proposed framework is developed based on Mask Region-based Convolutional Neural Networks (R-CNN) and extended to integrate the joint feature, body boundary, body orientation and occlusion condition together. In order to further improve the performance of the multi-person pose estimation, this paper proposes to organize the different information in serial multi-task models instead of the widely used parallel multi-task network. The proposed models are trained on the public dataset Common Objects in Context (COCO), which is further augmented by ground truths of body orientation and mutual-occlusion mask. Experiments demonstrate the performance of the proposed method for multi-person pose estimation and body orientation estimation. The proposed method can detect 84.6% of the Percentage of Correct Keypoints (PCK) and has an 83.7% Correct Detection Rate (CDR). Comparisons further illustrate the proposed model can reduce the over-detection compared with other methods.

## 1. Introduction

Human pose estimation is defined as the problem of the localization of human joints (also known as key points—elbows, wrists, etc.) in images or videos. It has become a highly concerned topic in computer vision. In addition, pose estimation also recently received significant attention from other research fields because of the valuable information contained in data of the human pose. 

### 1.1. Non-Deep Neural Network Approach

In the early stage of the pose estimation research, researchers focus on the simple scenario, and apply the background subtraction to extract the human silhouette from an image sequence. The model-based method enforces a pre-defined human model to be consistent with the extracted silhouette to estimate the human pose [1]. When the model-based method is performed in the multiple views, the silhouette and human pose in 3D can be extracted and estimated [2].

In addition to silhouette information, the temporal information in image sequence is often used to improve the performance of the pose estimation. One kind of these methods is based on detection and tracking, which detects a rough pose in the initial frame and tracks the pose in every continuous frame [3]. After that, the sophisticated model, such as the spatial–temporal Markov Random Field (MRF) model, is applied for pose estimation in image sequence [4]. A spatial–temporal constraint is also used for optimizing body-part configurations in videos [5]. These models are benefited from the position constraints of both intra-frame joints and inter-frame joints. 

Compared to image sequence based pose estimation, extracting a human pose from a single image is a more challenging topic. It requires to detect a human from variant backgrounds and estimate the pose in a large number of degrees of freedom using the limited information in the single frame only. For the single image based pose estimation, Pictorial Structures [6], Deformable Part Models (DPM) [7] and the integration between the two methods [8] are the famous techniques prior to deep neural networks. 

### 1.2. Deep Neural Network for Single Person Pose Estimation

In recent years, Deep Neural Networks (DNN) have achieved high performance and outperformed the conventional methods in many different tasks of computer vision. For example, the proposed Alexnet [9], Visual Geometry Group (VGG) Net [10], GoogLeNet [11] and ResNet [12] improve the accuracy of the object classification step by step. Fast Region-based Convolutional Neural Networks (R-CNN) [13], You Only Look Once (YOLO) [14] and Mask R-CNN [15] also become the new milestones of object detection and segmentation tasks, respectively. In addition, DNNs have been widely used for human pose estimation.

Pose estimation can be classified into single person pose estimation and multi-person pose estimation based on the number of humans that appear in the image. Toshev et al. [16] propose the DeepPose to estimate the pose of a single person from coarse to fine in a cascade DNN structure. However, two limitations exist in their method. Firstly, if the result of the initial pose estimation at the beginning of the cascade DNN is far from the true position, the system will not be able to correct the estimation because the cascade structure is a one-way flow. Secondly, this method only provides one prediction per image, which means there is no possibility to improve the prediction when additional information becomes available for the pose estimation. To address the first issue, Haque et al. [17] and Carreira et al. [18] propose to apply feedback structures to iteratively optimize the pose estimation. Their methods feed the estimated pose from the early iterations to the input end again and gradually refine the pose in the next iterations. As for the second problem, methods using probabilistic heatmaps are proposed as a solution. These heatmaps generated by Convolutional Neural Networks (CNN) turn the joint estimation problem into a pixel-wise classification in pyramid feature maps [19].

In addition, Wei et al. [20] build a convolutional pose machine network for single person pose estimation. Their method learns implicit spatial models via a sequential composition of convolutional architectures, because the easier-to-detect joint can provide strong cues for localizing the difficult-to-detect joint. Similarly, Newell et al. [21] present an hourglass module to capture information at every scale in order to combine features from different stages better. Wang et al. [22] propose a novel densely connected convolutional module-based convolutional neural network to estimate the pose of a single person. Wang et al. [23] apply the multi-scale feature pyramid module to further improve the performance of the deeply learned compositional model of human pose estimation. Chen et al. [24] propose to use generative adversarial networks to exploit the constrained human-pose distribution for improving single-person pose estimation. Szczuko proposes to localize single-person body joints in 3D space based on a single low resolution depth image [25].

### 1.3. Deep Neural Network for Multi-Person Pose Estimation

However, the pose estimation task becomes more complex in the case of multi-person. Firstly, assembling many homogeneous joints to different persons is a challenging issue. Secondly, occlusion caused by overlapping between multi-persons makes the detection of joint much more difficult. In order to deal with the multi-person pose estimation, the solutions are divided into two types: top-down approaches and bottom-up approaches.

In bottom-up approaches, firstly body part or joint detection is conducted, and then the accurate prediction of the number of people and their poses is performed by person clustering and joint labeling. Pishchulin et al. [26] propose a DeepCut method where Fast R-CNN [13] is used as a body part detector. Then the goal of the task becomes subset partitioning from a graph of all connections among each joint detected from the image, which turns the task into an Integer Linear Programming (ILP) problem. After that, they improve DeepCut by using deeper ResNet architectures [12] to enhance body part detectors and propose image-conditioned pairwise terms that assemble the proposals into a variable number of consistent body part configurations [27]. Kocabas et al. propose to estimate the multi-person pose using a pose residual network [28]. The proposed system is named as the MultiPoseNet and has real time performance. Furthermore, in order to achieve the goal of real-time pose estimation, Cao et al. present a new method using Part Affinity Fields (PAFs) [29], which is also known as OpenPose later. The proposed method firstly generates the joint positions of all of the people in the image by finding the local maximums of joint heatmaps. Then the method connects these joints gradually to construct the person pose structures. This method performs well for visible joints. However, there are many false positives for the occluded joints, because of its aggressive searching of joint positions. 

Although bottom-up approaches show a general advantage in runtime analysis, these approaches still suffer from problems that people in the image are in small scale or collusion, because detecting body parts before detecting the person itself becomes even difficult. In this case, top-down approaches could have better results by firstly detecting the person and then proceeding single-person pose estimation in each bounding box. Papandreou et al. [30] propose a method using a two-stage network that uses a person box detection system [31] as a bounding box detector, then predicts the pose of every single person in each bounding box. He et al. [15] also develop a multi-task method based on their former contribution [32], which can be implemented for pose estimation tasks.

### 1.4. Information Used for Human Pose Estimation

Joint feature is the most direct information for human pose estimation. In addition, human detection [30] and human segmentation [15] have been used for improving the accuracy of pose estimation. Furthermore, in order to handle the self-occlusion in pose estimation, Azizpour et al. [33] and Ghiasi et al. [34] learn templates for occluded versions of each body part. Rafi et al. [35] incorporate the context information of occluding objects to predict the locations of occluded joints. Haque et al. [17] implicitly learn the occlusion through a deep neural network and gave the output of a visibility mask of joints. In our previous work [36], orientation prediction is integrated with single person pose estimation.

Table 1 shows a comparative overview of the different previous frameworks. Non-deep neural network approaches require image sequence [1,2,3,4,5], or hand-crafted features and models [1,6,7,8] for pose estimation. In addition, the accuracy of non-deep neural network approaches is lower than the following deep neural network approaches [16,18]. The single person pose estimation works with the guarantee of only one person present in the image [16,17,18,19,20,21,22,23,24,25,35,36]. However, the scenes with multi-person are exceedingly common in our daily life. Thus, multi-person pose estimation frameworks are more universal for real-world applications. Current multi-person pose estimation approaches are the lack of effectively using occlusion and orientation information, and suffer from the false positive detection problem. By considering the above mentioned points, this paper proposes to integrate the body orientation and occlusion information into the multi-task learning framework for the multi-person pose estimation. In addition, this paper not only simply implements the widely used parallel multi-task network, but also develops the serial multi-task networks to reduce the false positive detection (over-detection) in the multi-person pose estimation. 

This paper has two contributions. First one is to propose a novel multi-task learning framework for the multi-person pose estimation. In the related works, the body segmentation, joint position estimation and joint visibility have been used for the human pose estimation. This paper proposes to add body orientation and mutual-occlusion information into the multi-task learning framework for the multi-person pose estimation. The second contribution of this research to propose to use the serial multi-task networks instead of the widely used parallel multi-task network for the multi-person pose estimation. To prove the advantage of serial multi-task networks, this paper compares the performance of the multi-task learning frameworks with different configurations for the multi-person pose estimation. In addition, for the purpose to train orientation and occlusion recognition tasks, our research team builds a dataset based on images from the Common Objects in Context (COCO) keypoints dataset by adding an orientation and occlusion mask as new annotations. The initial ideas of this paper have been published in our previous conference papers [37]. Compared to our previous conference publication, this paper gives more surveys about the related works. In addition, this paper compares the performance of the different versions of the developed pose estimation networks.

The rest paper is organized as follows: Section 2 describes the proposed pose estimation networks. Section 3 evaluates the performance of the proposed networks. Finally, Section 4 concludes this paper.

## 2. Deep Neural Network for Multi-Person Pose Estimation

Human pose estimation is a challenging task in computer vision. One of the difficulties is the occlusion problem, including both self-occlusion and mutual-occlusion by other objects. The main reason of self-occlusion is body orientation. For example, if a standing person is facing the right of the camera, his or her left body is probably occluded. On the other hand, the mutual-occlusions may happen due to arbitrary objects. Another difficulty of pose estimation is the left-right similarity problem because of the symmetry of the human body. For example, the left shoulder of a person in the back view is very similar to the right shoulder in the front view. Therefore, to solve these problems, this research proposed to incorporate the body orientation and occlusion condition into the pose estimation.

In this paper, Mask R-CNN [15] was chosen as a basic model to build our multi-task model, because the top-down region proposal network of Mask R-CNN makes it feasible to implement new tasks into the multi-task and the pose estimation task of Mask R-CNN also has a certain space to improve. In the proposed models, body boundary, body orientation and occlusion condition and joint position estimation tasks were integrated in order to improve the accuracy of multi-person pose estimation. Body boundary can be represented as the silhouette. Body orientation is the human body direction relative in the camera space. The occlusion condition in the multi-person case can be separated into two situations: self-occlusion and mutual-occlusion. The more detailed information of these tasks will be described in the following subsections. 

**Figure 1:**
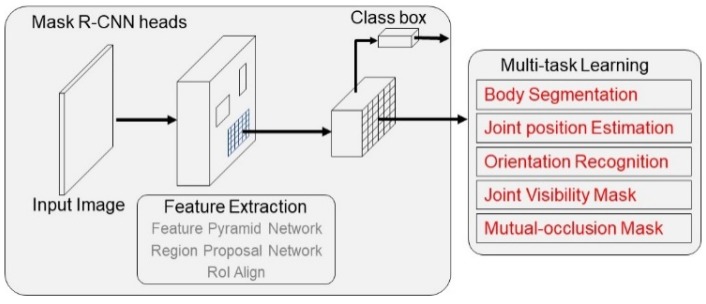
The framework of the proposed multi-task based pose estimation model.

The system framework of the proposed method is illustrated in Figure 1. The input image would firstly be resized to 1024 × 1024 in the preprocessing step, then input into a Mask R-CNN layer heads for feature extraction, which consists of the feature pyramid network, region proposal network and a Region of Interest (RoI) align layer at last to generate RoI features. In addition, the two-stage object detection and classification branch in the original Mask R-CNN was also conducted in this layer heads. In fact, this paper also adopted the detection and classification branch of Mask R-CNN to obtain the RoI feature of each human. RoI features were then fed into the multi-task network. The multi-task learning part was the focus of this paper, and the details of the multi-task learning part were explained in the next subsections. 

### 2.1. Parallel Multi-Task Network for Pose Estimation

**Figure 2:**
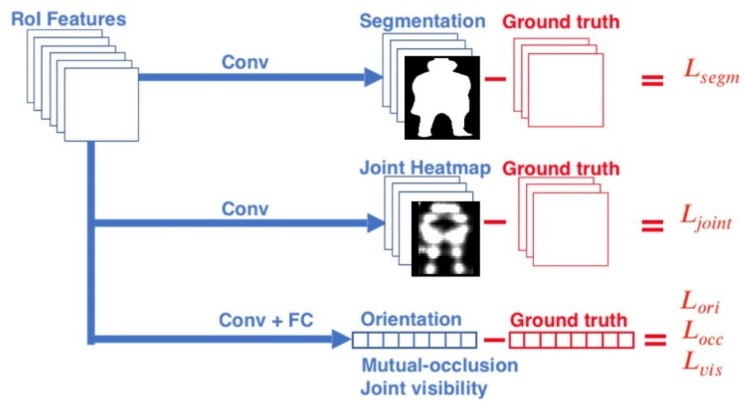
Architecture of the proposed parallel multi-task training network.

In this paper, the widely used parallel multi-task network was firstly chosen to integrate the multi-information. The architecture of the parallel multi-task network is illustrated in Figure 2. The network has three branches: body segmentation branch, joint position estimation branch and occlusion-orientation branch. The three branches output five multi-task results: segmentation, joint heatmap, orientation, joint visibility and mutual occlusion. In the training step, the five multi-task results were used for calculating the value of loss function. In this paper, the multi-task loss *L* was defined as the sum of the loss from each task:

(1) $$L=L_{segm}+L_{joint}+L_{ori}+L_{vis}+L_{occ},$$

where, $L_{segm},$ $L_{joint},$ $L_{ori},$ $L_{vis}$ and $L_{occ}$ denote the loss function for the task segmentation, joint heatmap estimation, orientation estimation, joint visibility and mutual occlusion estimation, individually. The details of each loss function will be explained in the next subsections. 

#### 2.1.1. Body Segmentation Branch

**Figure 3:**
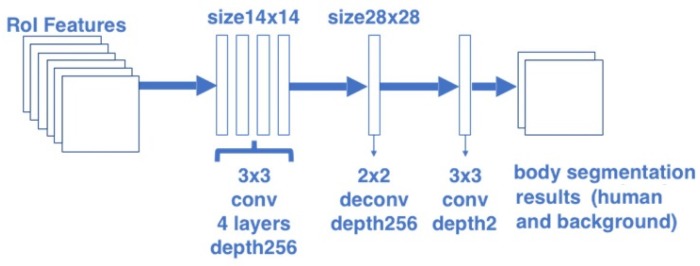
Architecture of the body segmentation branch.

To build the body segmentation branch, this paper borrowed the segmentation part of Mask R-CNN and changed the number of object classes into 2 (person and background). The architecture of this branch is illustrated in Figure 3. The body segmentation branch predicts masks for each RoI using a Fully Convolutional Networks (FCN) [38]. This branch consists of a stack of four convolutional layers with kernel size 3 and depth 256, followed by a deconvolution layer and a final convolutional layer with kernel size 3. In this branch, the 14 × 14 size RoI features are firstly passed into the segmentation head, and finally converted to an output with the resolution 28 × 28 and depth 2. This structure allows each layer in the branch to maintain the explicit object spatial layout without collapsing it into a vector representation that lacks spatial dimensions. 

The segmentation loss $L_{segm}$ is defined as a cross entropy between ground truth and segmentation result with considering the class label of the segmentation result:

(2) $$L_{segm}=-\frac{1}{N_{RoI}}\sum_{i=1}^{N_{RoI}}\left(Segm_{i}*class_{i}\right)*\log Segm_{i}^{\prime },$$

where *i* is the index of RoI. $class_{i}$ is the ground truth class label. $Segm_{i}^{\prime }$ is the segmentation ground truth and $Segm_{i}$ denotes the segmentation result.

#### 2.1.2. Joint Position Estimation Branch

**Figure 4:**
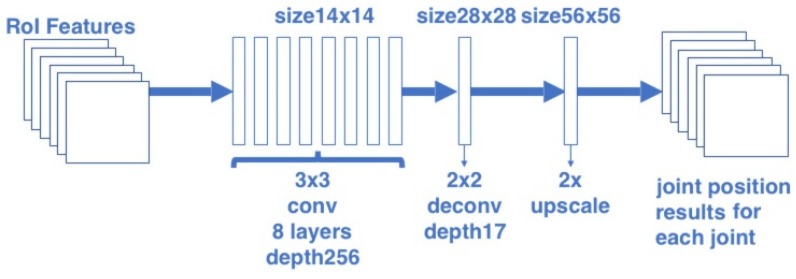
Architecture of the joint position estimation branch.

In the joint position estimation branch, the joint position is considered as a “one-hot” mask. The joint position estimation branch uses the architecture similar to the body segmentation branch for predicting $N_{joint}$ masks. Each mask is corresponding to one of $N_{joint}$ joint types (e.g., left shoulder, right elbow). The architecture of the joint position estimation branch is shown in Figure 4. Similar to the body segmentation task, this paper used the FCN structure to output these $N_{joint}$ masks. For the reason that the joint is much smaller in each RoI, the output joint mask size is set twice as the segmentation mask in order to give accurate results. In this branch, the 14 × 14 RoI features are firstly input into a stack of eight convolutional layers with kernel size 3 and depth 256, then passed through a deconvolution layer with kernel size 2 and a bilinear interpolation up-scaling layer, finally converted to an output with the resolution 56 × 56 and depth $N_{joint}$. 

The joint position loss $L_{joint}$ is defined as Equation (3).

(3) $$L_{joint}=-\frac{1}{17N_{ROI}}\sum_{i=1}^{N_{ROI}}\sum_{j=1}^{N_{joint}=17}\mathrm{Softmax}(K_{ij})*\log K_{ij}^{\prime }$$

where the number of joint $N_{joint}$ is set to 17 in this research. $K_{ij}^{\prime }$ is the ground truth, $K_{ij}$ is the joint position estimation result. Instead of the pixel-wise cross entropy used in body segmentation branch, this paper used one-hot label ground truth where only one correct joint position is true in the joint position loss function. This loss function can force the network to output a probability contribution where only one pixel in the mask gives the peak value after softmax operation.

#### 2.1.3. Orientation-Occlusion Branch

**Figure 5:**
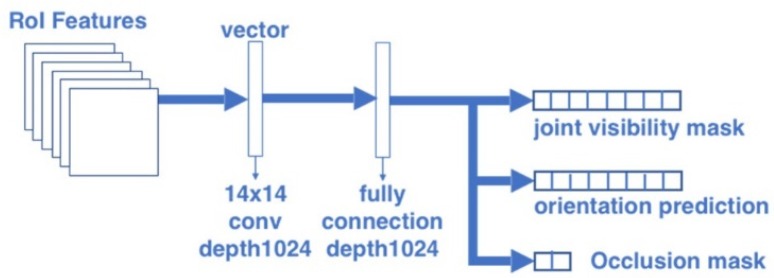
Architecture of the orientation-occlusion branch.

**Figure 6:**
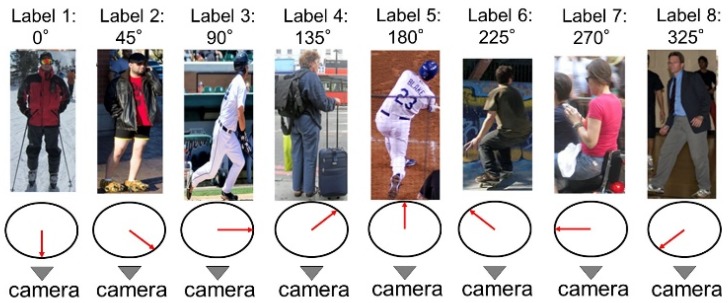
The representations of body orientations.

The architecture of the orientation-occlusion branch is shown in Figure 5. This branch consists of a convolutional layer with kernel size 14 × 14 and depth 1024, followed by a fully connected layer. The body orientation is a kind of global information of pose configuration. The body orientation is useful for solving both the self-occlusion problem and left-right similarity problem. For example, if we know a person is facing to right, the body orientation indicates the occlusion of his or her left body. Similarly, if a person is facing the camera, his or her right shoulder is probably on the left side of the image. Our previous research [36] suggests that body orientation could be defined as 8 directions, as shown in Figure 6. When the body is defined as 8 directions, the prediction result of the body orientation can be a vector format with 8 elements.

The term visibility mask is introduced by Haque et al. [17]. However, they only applied the visibility mask in the top view images for self-occlusions. This research extends the application of the visibility mask for more flexible view angles and for both self-occlusion and mutual-occlusion by objects. In this research, the joint visibility mask is a 17-dimensional vector to indicate the visibility of each joint. In addition, the occlusion mask is defined as a 2-dimensional vector. The combination of occlusion mask and joint visibility mask can explain the reason for the invisible joints: only self-occlusion or self-occlusion plus mutual-occlusion. The loss of these 3 outputs are defined as the cross entropy with softmax:

(4) $$L_{ori}=-\frac{1}{N_{ROI}}\sum_{i=1}^{N_{ROI}}\mathrm{Softmax}(Ori_{i})*\log Ori_{i}^{\prime },$$

(5) $$L_{vis}=-\frac{1}{N_{ROI}}\sum_{i=1}^{N_{ROI}}\mathrm{Softmax}\left(Vis_{i}\right)*\log Vis_{i}^{\prime },$$

(6) $$L_{occ}=-\frac{1}{N_{ROI}}\sum_{i=1}^{N_{ROI}}\mathrm{Softmax}\left(Occ_{i}\right)*\log Occ_{i}^{\prime },$$

where the terms $Ori_{i}{}^{\prime }$, $Vis_{i}{}^{\prime }$ and $Occ_{i}{}^{\prime }$ are the ground truth labels of body orientation, joint visibility and mutual-occlusion, respectively. The terms $Ori_{i}$, $Vis_{i}$ and $Occ_{i}$ are the estimation results of body orientation, joint visibility and mutual-occlusion output from this branch.

### 2.2. Serial Multi-Task Pose Estimation Network

**Figure 7:**
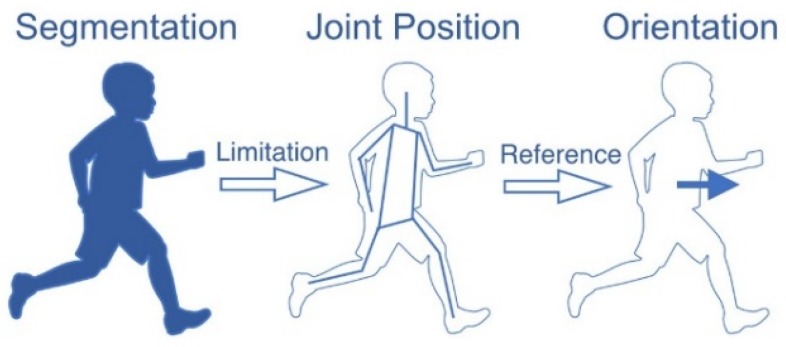
An example of the relationships between different tasks.

In the parallel multi-task network, the information that can only be shared between different tasks is the RoI features extracted from the Feature Pyramid Network (FPN) and RoI align layer. Especially for the body segmentation task and joint position estimation task, the RoI features is hardly affected by the activation of ground truth label during backpropagation because of the deep convolutional networks. Actually, the information used in human pose estimation is strongly related to each other. Figure 7 shows an example of the relationship between the different tasks. The body segmentation describes the boundary of the human body, it can be a constraint for the joint position estimation. In addition, joint positions are helpful to model the structure of the human pose, which can be a strong reference for body orientation estimation. This is not the only way that this information is related, there are more combinations that the different tasks can benefit from each other. In order to take advantage of these relationships and enhance the connections between different tasks, this paper proposes to use the serial multi-task networks instead of the widely used parallel multi-task network for the multi-person pose estimation.

**Figure 8:**
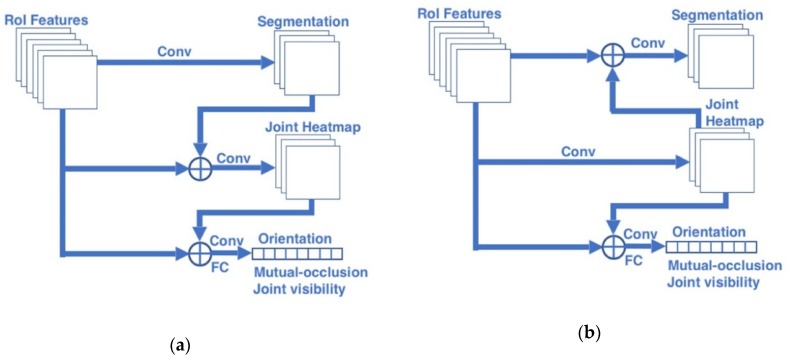
Proposed two serial multi-task networks. (**a**) Serial multi-task network with “Segmentation→Joint” connection; (**b**) Serial multi-task network with “Joint→ Segmentation” connection.

Figure 8 shows two forward passing architectures of the proposed serial multi-task networks. Since the orientation and occlusion outputs are vectors that have a smaller amount of data compared to the segmentation mask and joint heatmap, the orientation-occlusion branch is set as the last output layer in this research. For the rest two branches, this paper proposed two models. In the first model, the body segmentation branch was configured at first and the output of segmentation branch was fed into the joint position estimation branch (as shown in Figure 8a). In the other model, the results of the joint position estimation were directly input into other two branches (as shown in Figure 8b). In both two serial multi-task networks, the joint position estimation branch is connected to the orientation-occlusion branch because the joint position is a better reference for the prediction of body orientation than the body segmentation. 

**Figure 9:**
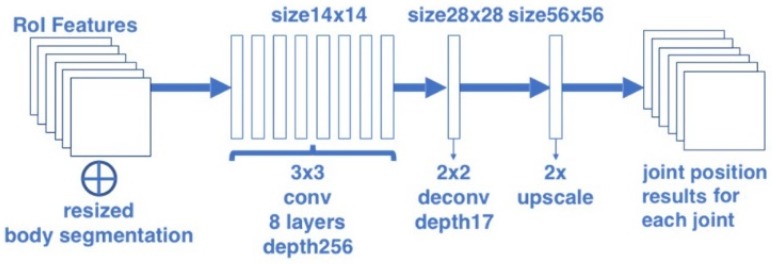
Joint position estimation branch combined with body segmentation (for Figure 8a).

As shown in Figure 8a, the output of the body segmentation branch is passed into the joint position estimation branch in the first serial model. Figure 9 illustrates the new architecture of the joint position estimation branch combined with body segmentation. In this new architecture, the body segmentation output (resized to 14 × 14) was added into the RoI features to make the body segmentation be referred into the convolutional process of the joint position estimation.

**Figure 10:**
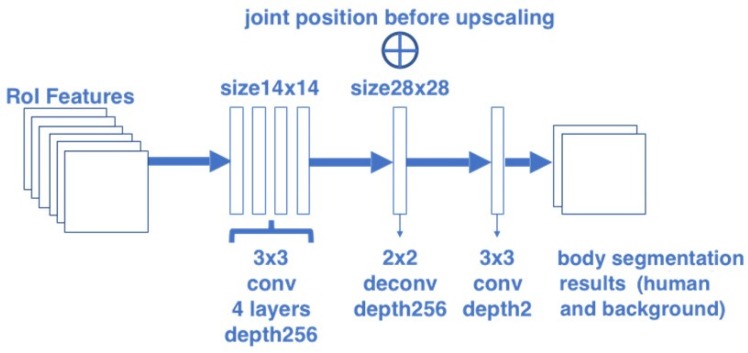
Body segmentation branch combined with joint position (for Figure 8b).

In the second serial model, joint position estimation results were input into the body segmentation branch, as shown in Figure 8b. This model added the intermediate result of the joint position estimation (before the last up-scaling layer (size 28 × 28)) into the body segmentation branch, as shown in Figure 10. 

**Figure 11:**
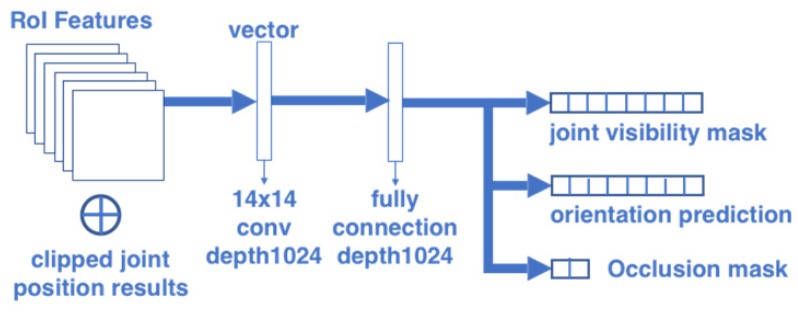
Orientation-occlusion branch combined with joint position (for Figure 8a,b).

In addition, both two serial models have a connection between the joint position estimation branch and orientation-occlusion branch. Figure 11 shows how to combine the joint position estimation result with the orientation-occlusion branch. This research clips the joint position results to be consistent with the size of RoI features. In this way, the clipped joint position features and RoI features are input into the fully connected network together. The performance of the two serial models will be presented in Section 3.

## 3. Results

This section will firstly describe the details of the training dataset, and then demonstrate the evaluations for the proposed models and comparison with other methods.

### 3.1. COCO Keypoint Dataset

**Figure 12:**
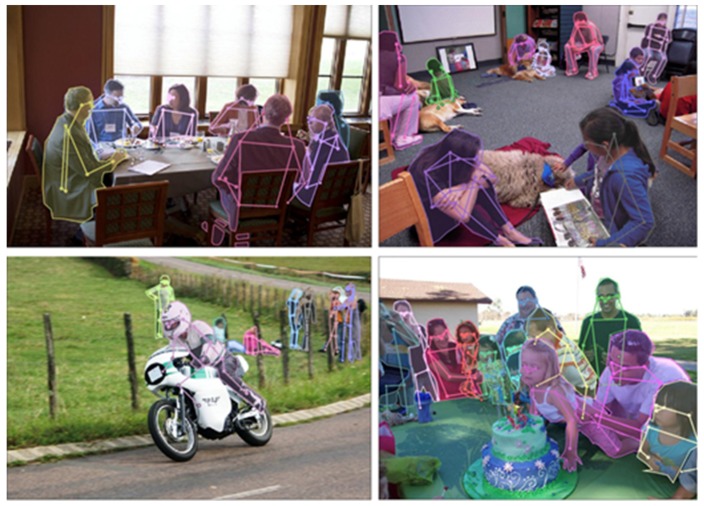
Demonstrations of Common Objects in Context (COCO) Keypoint Dataset 2017.

In this paper, the COCO keypoint dataset [39] was used for training and evaluation. COCO (Common Objects in Context) dataset is an open dataset built by Microsoft and Facebook, etc., which has a large volume of images for general object detection and segmentation tasks. Keypoint detection is one of the tasks in the COCO dataset, which requires localization of person keypoints in challenging, uncontrolled conditions. COCO keypoint dataset is the largest 2D pose estimation dataset and has been widely adopted by other multi-person 2D pose estimation algorithms. In addition, the images of the COCO keypoint dataset were captured in human’s daily life, such as a party, meeting room and sport field. The images include multi-person with different scales, orientations, occlusions and postures. Figure 12 gives a demonstration of this task, each person in the image has a set of annotations including segmentation, class name (in this task it is “person”), bounding box position, the number of labeled keypoints and a list of body joint information (labeled in the format (*x,y,v*) with 17 joints, where *x,y* is the location of joint and *v* is the visibility of joint that *v* = 0 means not labeled, *v* = 1 means labeled but not visible and *v* = 2 means labeled and visible). The proposed models were trained on the COCO Keypoint Dataset 2017, which includes 56,599 images for training. In the training, the instances with less than three joints were excluded in order to teach the model to learn the whole body structure better and accelerate the converge of the training process.

### 3.2. Extended Sub Dataset with Mutual-Occlusion and Body Orientation

Since this paper added the mutual-occlusion estimation and orientation recognition task in the proposed models, which require the annotations that do not exist in the COCO keypoint dataset, our research team built a sub dataset for training these tasks and evaluation of the models. The subdataset included 2306 images from the training dataset of the COCO keypoint dataset and 260 images from the validation dataset in the COCO keypoint dataset. The images include over 8000 human instances in different sizes and situations. The 2306 images are used in training, and the 260 images were used for evaluation.

The label of body orientation follows the definition shown in Figure 6. This paper divided the horizontal space into eight parts with each part of 45 degrees to label different body orientations. However, because some instances are hard to be categorized into certain orientation, for this reason, a label “9” was added to deal with those instance orientations that were hard to distinguish. It means that the prediction result of the body orientation was a vector format with nine elements in this research.

**Figure 13:**
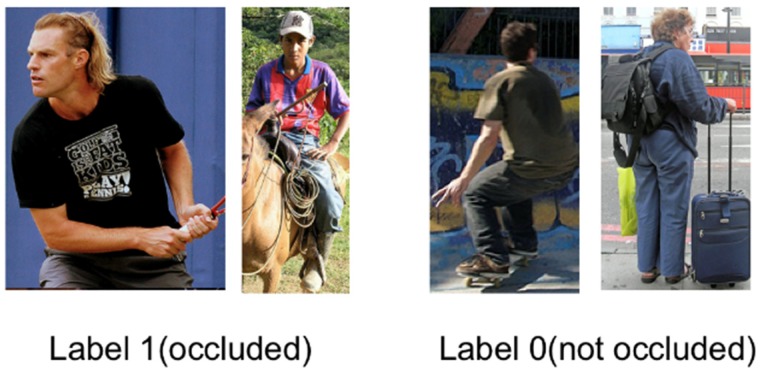
The label system for the mutual-occlusion mask in our subdataset.

The label system for the mutual-occlusion mask is shown in Figure 13. Unlike the joint visibility mask that is a binary mask for each joint of each instance, the mutual-occlusion mask is defined as a binary mask for the whole instance in one bounding box. It is a classification label that categorizes the person in each bounding box into two classes: occluded and not occluded. To distinguish this mutual-occlusion label from self-occlusion, our research team only labeled the instances that were cropped by the edge of the image or occluded by another person (or object) with Label 1 (occluded). For the instance that has self-occlusion due to its orientation, we labeled it as Label 0 (not occluded). 

### 3.3. Dataset for Training and Evaluation

In the research, all the models (will be presented in Table 2, Table 3, Table 4, Table 5, Table 6 and Table 7) were trained on the training dataset of COCO Keypoint Dataset 2017. More specifically, the proposed models (both the parallel and serial models) were first “pre-trained” on the training dataset of the COCO keypoint dataset for the body segmentation task, joint position estimation task and joint visibility mask task. After that, 2306 images and their annotation (including segmentation, joint position, joint visibility mask, mutual-occlusion and body orientation) in the extended subdataset were reused to retrain all the branches of our proposed models. This retraining processing started from the pretrained networks. For other methods (Openpose [29] and MultiPoseNet [28]) adopted in the comparisons, the models are trained on the same training dataset.

In the evaluation for joint position estimation (will be presented in Table 2, Table 5 and Table 6), all the models (including both our proposed models and models in other methods) were evaluated on the validation dataset of the COCO keypoint dataset. 

In the evaluation for body orientation recognition (will be presented in Table 3 and Table 4), the models (including both the parallel models and serial models) were evaluated on 260 images in the extended subdataset. The 260 images had orientation annotations labeled by our research team.

### 3.4. Evaluation for Joint Position Estimation

To evaluate the performance for the joint position estimation, this paper used the Percentage of Correct Keypoints (PCK), which is a widely used evaluation metric. The correct keypoint is defined as the predicted joint whose distance from the true joint is less than a given threshold. In this research, the threshold used in the PCK calculation was set as 0.1 × bounding box height. PCK can evaluate how many percentages of keypoints can be correctly detected. Calculation of PCK can be expressed as Equation (7).

(7) $$\mathrm{Percentage}\;\mathrm{of}\;\mathrm{Correct}\;\mathrm{Keypoints}=\frac{\sum \left(Joint_{detected}\cap Joint_{groundtruth}{|}_{vis>0}\right)}{\sum \left(Joint_{groundtruth}{|}_{vis>0}\right)},$$

where $Joint_{detected}\cap Joint_{groundtruth}$ is the joints correctly detected by the network and $Joint_{groundtruth}{|}_{vis>0}$ is the visible joints labeled in the ground truth annotations. In the evaluation, the PCK of the joints was calculated by considering visibility > 0.

The evaluation results and comparisons between the different proposed models are shown in Table 2. In Table 2, “Joint+Segm” represents a parallel multi-task model before training on the occlusion and orientation subdataset, “Segm→Joint” represents the serial multi-task model that inputs body segmentation results into the joint position estimation branch, “Joint→Segm” represents the serial multi-task model that inputs joint position estimation results into the body segmentation branch, and “with Occ&Ori” represents models trained on the occlusion and orientation subset.

**Figure 14:**
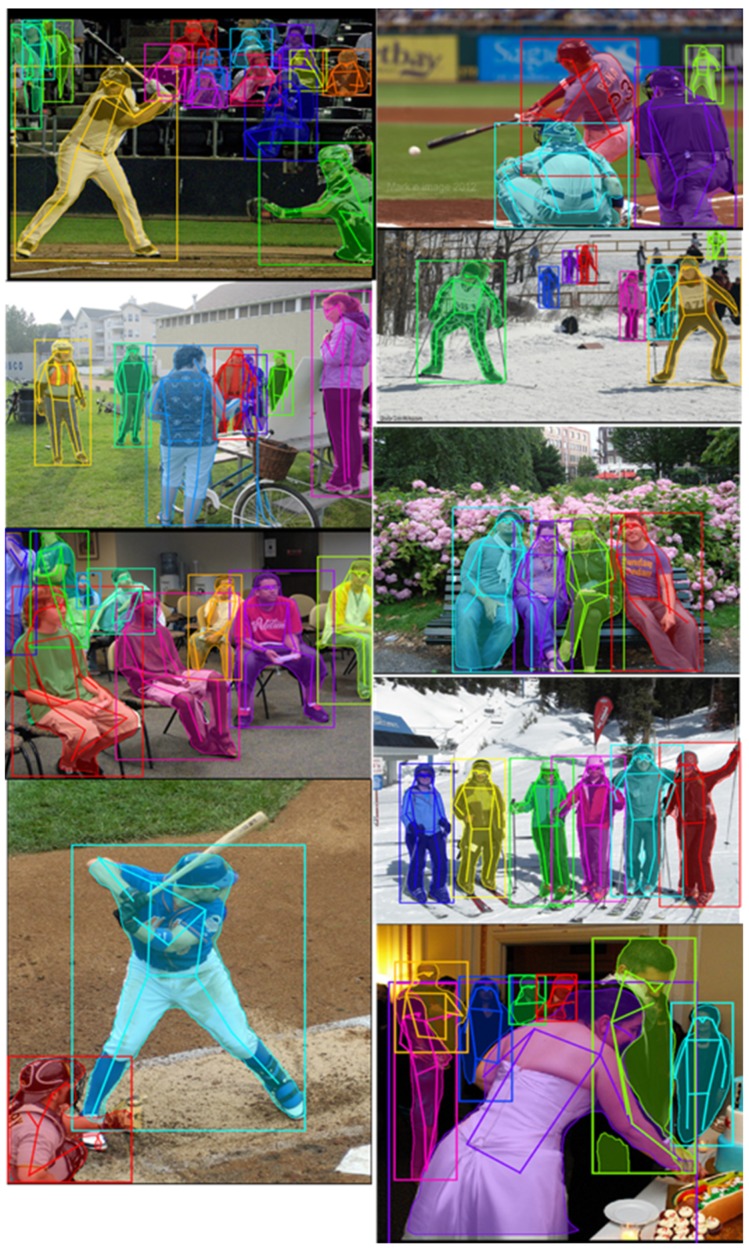
Body segmentation and joint position estimation results on the COCO dataset.

**Figure 15:**
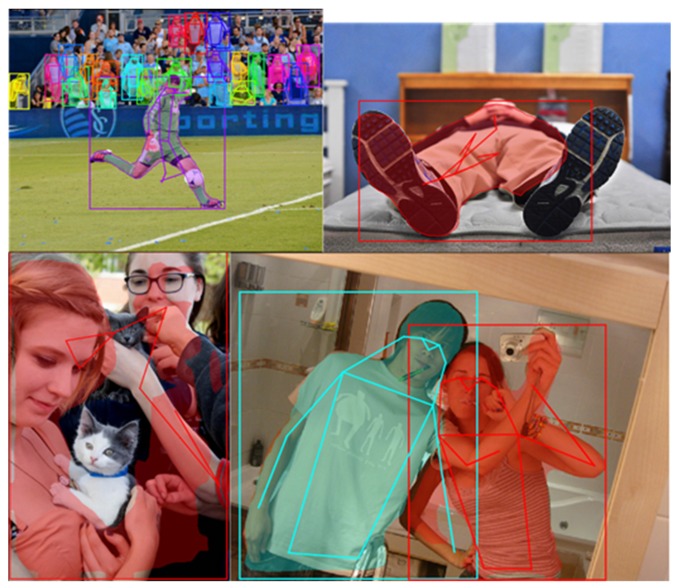
Some incorrect joint position estimation results of the proposed model.

The evaluation results show that after training on our occlusion and orientation subdataset, the accuracy of each model had an overall increase on each joint. The proposed serial multi-task models obtained higher accuracy than the parallel multi-task model. In addition, the serial model starts from the joint position estimation branch had the best performance. Some results of this model on multi-person images are shown in Figure 14. The examples of incorrect joint position estimation results are shown in Figure 15, where the image on the left top is an example that our model did not separate the two ankles but activated them at the same time, which led to the low accuracy of the ankle. For the human instances with an uncommon pose (image on the right top of Figure 15), our model also could not make a correct estimation. The two images on the bottom show examples where the body segmentation and joint position estimation gave incorrect results together, which is a problem of the multi-task network that we need to solve in the future. 

### 3.5. Evaluation for Orientation Estimation

This paper also evaluated the orientation recognition performance of the proposed models, which was also a widely used benchmark for human pose estimation. Table 3 shows the accuracy of the orientation recognition task of the proposed models. In the first evaluation, the predicted orientation result was correct if the result was exactly the same as the ground truth orientation. We could see the benefit after we input joint position estimation results into the occlusion-orientation branch. The serial model starts from joint position estimation had the best overall accuracy, where we thought body orientation estimation benefitted from the increase of joint position accuracy.

**Figure 16:**
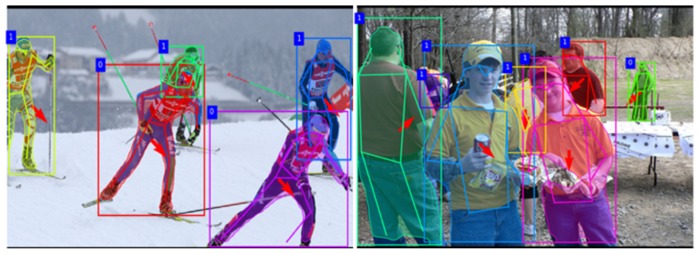
Examples of the results for orientation recognition.

In addition, this paper adopted a compatible principle that allows a neighboring error for the body orientation estimation in the second evaluation. As shown in Table 4, the proposed models show more satisfying performance on the orientation prediction. For the normal eight orientations, the two serial models obtained a similar high accuracy. However, for the other orientation (Label 9), the serial model starts from the joint estimation branch had a better recognition rate than other models. Figure 16 visualizes several results of the whole multi-task outputs of the proposed model, where the red arrow in the center of each bounding box shows the body orientation recognition result, and the number with a blue background on the left top of bounding box represents the mutual-occlusion condition of each person (1 is mutual-occluded and 0 is not mutual-occluded).

### 3.6. Comparison with Other Methods

In order to demonstrate the effectiveness of the proposed method, this paper compared the proposed multi-task models with other pose estimation methods. The comparison among our models, OpenPose [29] and MultiPoseNet [28], is shown in Table 5. Our serial model starts from joint position estimation obtained higher overall PCK than MultiPoseNet, which benefits from the higher accuracy of the face parts. For the whole-body joints without face parts, our model gave similar PCK as MultiPoseNet. The OpenPose performed higher PCK by taking advantage of its bottom-up architecture and part affinity filed network. However, we found that OpenPose had an obvious over-detection tendency on the “not joint” objects because it did not use the bounding box to limit the area of convolution. 

In order to demonstrate the ability to make correct joint detections, this paper defined a Correct Detection Rate (CDR) to evaluate the over-detection tendency, which can be expressed as:

(8) $$\mathrm{Correct}\;\mathrm{Detection}\;\mathrm{Rate}=\frac{\sum \left(Joint_{detected}\cap Joint_{groundtruth}{|}_{vis>0}\right)}{\sum \left(Joint_{detected}\right)},$$

where $Joint_{detected}$ is the joints detected by the network and $Joint_{groundtruth}$ is the joints labeled in the ground truth annotations. When a joint out of annotation is detected, it will be counted as an over-detection. The comparison between our proposed method and other methods using CDR is shown in Table 6. 

**Figure 17:**
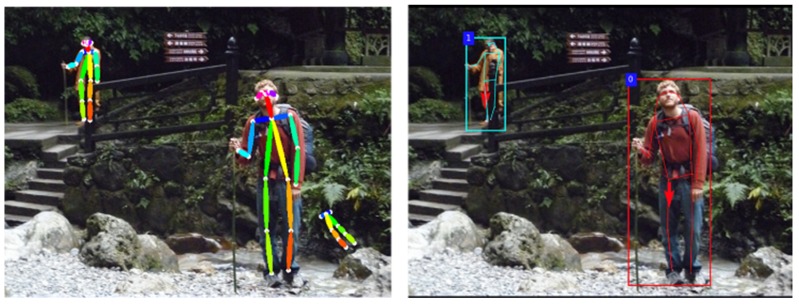
Examples of human pose estimation results generated by OpenPose [29] (left) and the proposed model (right).

This paper evaluated the performance of the human pose estimation algorithms by considering both PCK and CDR. PCK denotes the percentage of correct keypoints and CDR represents the over-detection tendency. Firstly, the proposed model (Joint→Segm with Occ&Ori) outperformed the MultiPoseNet in the comparisons using both PCK and CDR. Secondly, the proposed model (Joint→Segm with Occ&Ori) achieved 84.6% PCK, it was slightly worse than 85.7% of OpenPose in the PCK comparison, as shown in Table 5. However, the proposed model (Joint→Segm with Occ&Ori) had 83.7% CDR, which was much better than the CDR of OpenPose 71.2%, as shown in Table 6. In order to visualize the effect of the over-detection, Figure 17 shows the human pose estimation results generated by OpenPose (left) and the proposed model (Joint→Segm with Occ&Ori; right). It is clearly seen that the result of OpenPose had an over-detection on the right bottom of the left figure. In fact, the low CDR value of the OpenPose method was caused by this kind of over-detection. By analyzing the PCK and CDR, we could understand that OpenPose used an aggressive manner in the detection of the joint position to maintain the higher PCK value, at the same time generated more over-detections. On the contrary, our proposed model dramatically reduced the over-detections and achieved the competitive result on PCK.

After the evaluation of the accuracy, the comparison was conducted for the processing speed. The processing speed of each model was calculated by averaging the processing time for each image used in joint position estimation. In the comparison for the processing speed, the processing time of different models were calculated by using the same images, and all experiments were performed on a system with an i7-8700K CPU and Nvidia 1080Ti GPU. The processing time of the parallel multi-task model, the serial multi-task models and other methods are summarized in Table 7. The parallel multi-task model with occlusion and orientation branches spends 1.16 s per image, and both two serial multi-task models need longer processing time than the parallel multi-task model, because later subnetworks need to wait for the earlier sub-networks to finish the process in the serial multi-task models, e.g., as shown in Figure 8b, the joint position estimation branch (label as “Joint Heatmap”) should finish the calculation completely, then the segmentation branch and orientation-occlusion branch can use the Joint Heatmap to finish the processing. Thus, this serial framework increased the processing time. In addition, our proposed models had longer processing time than the conventional methods [28,29]. Overall, our proposed model had better accuracy but required more processing time compared to the conventional methods.

This proposed algorithm could be used for some applications that need high accuracy but do not require real-time processing speed, e.g., our previously published works [36,40] propose to analyze the customer pose for marketing. The customer behavior analysis is one of the most concerned topics for retailers because the customer behavior information can indicate the customer interest level to the product in the stores and is helpful to increase the commercial benefit. By checking the body orientation, head orientation and pose interactions between merchandise (such as touching, taking items, returning to shelf or putting into basket), these customer behaviors can reveal their interest level to the merchandise. The proposed methods in this paper can be used to analyze the recorded images by the surveillance camera in the store. High accuracy of the pose estimation is expected for the purpose of the commercial benefit. In addition, the real-time processing speed is not required because the customer behavior analysis could be post-processing. Thus, the customer behavior analysis application is one of the examples that the proposed method can be applied for.

This research has proven that integrating body orientation and occlusion information into the pose estimation multi-task network could improve accuracy. In the future, the temporal information between frames will be considered and utilized for the multi-person pose estimation in video. In addition, the temporal information is expected to be a solution to improve the low accuracy issue for ankle joints.

## 4. Conclusions

This research presented a multi-person pose estimation method using multi-task deep learning networks. The proposed network model is the extended multi-task network based on a Mask R-CNN layer heads, and it consists of five tasks: (1) joint position estimation, (2) body segmentation, (3) joint visibility mask, (4) body orientation recognition and (5) mutual-occlusion mask, the five tasks are separated into three branches: body segmentation branch, joint position estimation branch and orientation-occlusion branch. This paper first built a parallel multi-task network with each task separately. In order to strengthen the connections between different tasks, this paper further proposed two serial multi-task models. In the evaluation step, this paper first evaluated the accuracy of the joint position estimation and orientation recognition ability of the proposed models. In addition, this paper compared the accuracy of the proposed model with other pose estimation methods by considering two criterions PCK and CDR. The proposed method could detect 84.6% PCK and had 83.7% CDR. Our proposed model could reduce the over-detection compared with other methods.

There are still some problems that need to be solved in the proposed models such as the low accuracy of ankle joints. In the future, we would like to continue improving this model by using the temporal information between frames for multi-person pose estimation in the video.

## Figures and Tables

**Table 1:** A comparative overview between the different previous frameworks.

Category		Method	Limitation
Non-Deep Neural Network Approach		Silhouette for pose [1,2] Spatial + temporal information for pose [3,4,5] Pictorial and part structure model for pose [6,7,8,33,34]	Requirement of image sequence, hand-crafted features and models Lower accuracy compared to deep neural network approaches
Deep Neural Network Approach	Single Person Pose Estimation	DeepPose [16], Feedback for pose [17,18] CNN + graphical model for pose [19] Convolutional pose machines [20] Stacked hourglass networks for pose [21] Densely connected convolutional module for pose [22] Multi-scale compositional models for pose [23] Generative adversarial network for pose [24] Pose in depth image [25] Semantic occlusion model for pose [35] Orientation for pose [36]	Limitation of only single person pose in one image
	Multi-Person Pose Estimation	DeepCut [26] DeeperCut [27] MultiPoseNet [28] Openpose [29] Two stages (Faster RCNN detector + fully convolutional ResNet) for pose [30] Mask-RCNN [15]	Lack of effectively using occlusion and orientation information False positive detection (over-detection)

**Table 2:** Evaluation for joint position estimation of proposed models using the Percentage of Correct Keypoints (PCK; %).

	Nose	Eye	Ear	Shoulder	Elbow	Wrist	Hip	Knee	Ankle	Overall
Joint+Segm	89.3	90.8	90.0	82.6	77.1	73.4	76.7	74.2	72.9	80.8
Joint+Segm With Occ&Ori	90.3	91.6	90.0	84.1	78.4	75.6	75.9	75.8	73.4	82.7
Segm→Joint	89.6	89.9	88.6	84.4	81.8	77.0	79.0	78.2	73.7	82.4
Segm→Joint With Occ&Ori	94.1	94.1	93.3	85.5	82.0	75.2	78.4	77.5	73.9	83.7
Joint→Segm	93.5	93.7	92.8	85.5	81.4	76.1	78.2	76.6	73.6	83.5
Joint→Segm With Occ&Ori	93.7	93.8	93.2	86.9	82.6	77.8	80.5	78.9	74.1	84.6

**Table 3:** Accuracy of orientation recognition in a strict principle (%).

Label	1	2	3	4	5	6	7	8	9
Degree	0	45	90	135	180	225	270	315	-
Joint+Segm with Occ&Ori	40.2	47.4	58.7	38.6	34.5	38.1	63.1	38.6	48.1
Segm→Joint with Occ&Ori	79.2	50.0	67.7	46.4	80.1	62.3	79.0	42.3	49.4
Joint→Segm with Occ&Ori	88.6	47.2	58.8	50.0	85.9	54.7	75.6	47.1	77.1

**Table 4:** Accuracy of orientation recognition when a neighbor error allowed (%).

Label	1	2	3	4	5	6	7	8	9
Degree	0	45	90	135	180	225	270	315	-
Joint+Segm with Occ&Ori	91.7	95.8	93.6	86.7	79.0	89.2	93.7	88.7	48.1
Segm→Joint with Occ&Ori	98.2	98.4	100	90.9	83.6	94.4	93.3	94.7	49.4
Joint→Segm with Occ&Ori	98.1	98.5	99.2	90.9	94.7	92.0	93.2	93.8	77.1

**Table 5:** Comparison between proposed model and other methods using PCK (%).

	Nose	Eye	Ear	Shoulder	Elbow	Wrist	Hip	Knee	Ankle	Overall
OpenPose [29]	92.2	92.1	92.3	89.5	83.9	74.2	83.3	83.5	80.8	85.7
MultiPoseNet [28]	88.9	88.5	88.9	84.5	82.0	79.6	78.6	79.7	78.0	83.2
Joint→Segm WithOcc&Ori	93.7	93.8	93.2	86.9	82.6	77.8	80.5	78.9	74.1	84.6

**Table 6:** Comparison between proposed models and other methods using Correct Detection Rate (CDR; %).

	Nose	Eye	Ear	Shoulder	Elbow	Wrist	Hip	Knee	Ankle	Overall
OpenPose [29]	72.2	70.5	66.2	74.8	69.3	72.8	72.7	74.8	66.5	71.2
MultiPoseNet [28]	85.6	81.1	77.6	88.3	82.4	80.5	83.7	80.3	71.0	81.2
Joint+Segm with Occ&Ori	82.7	80.4	75.2	86.0	80.0	78.4	88.4	87.7	82.9	82.4
Segm→Joint with Occ&Ori	89.3	86.6	79.2	85.2	77.7	77.8	77.8	85.3	82.7	82.4
Joint→Segm with Occ&Ori	84.7	86.6	80.6	85.2	82.3	81.4	85.8	87.8	79.3	83.7

**Table 7:** Comparison for processing time of proposed models and other methods.

Method	Processing Time (Second)
OpenPose [29]	0.26
MultiPoseNet [28]	0.05
Joint+Segm with Occ&Ori (Parallel model)	1.16
Segm→Joint with Occ&Ori (Serial model)	1.21
Joint→Segm with Occ&Ori (Serial model)	1.23
